# Formulation, Cellular Uptake and Cytotoxicity of Thymoquinone-Loaded PLGA Nanoparticles in Malignant Melanoma Cancer Cells

**DOI:** 10.2147/IJN.S269340

**Published:** 2020-10-20

**Authors:** Wisam Nabeel Ibrahim, Luqman Muizzuddin Bin Mohd Rosli, Abd Almonem Doolaanea

**Affiliations:** 1 Department of Biomedical Sciences, College of Health Sciences, QU Health, Qatar University, Doha, Qatar; 2 Department of Pharmaceutical Technology, Faculty of Pharmacy, International Islamic University Malaysia, Kuantan 25200, Malaysia

**Keywords:** microencapsulation, thymoquinone, PLGA, nanoparticles, melanoma

## Abstract

**Introduction:**

Thymoquinone (TQ) is the main active compound extracted from *Nigella sativa* a traditional herb with wide therapeutic applications and recognizable anticancer properties. This study aimed to formulate and characterize TQ-nanoparticles using PLGA as a biocompatible coating material (TQ-PLGA NPs) with the evaluation of its therapeutic properties in human melanoma cancer cells.

**Methods:**

The TQ-PLGA NPs were prepared and characterized for size, zeta potential, encapsulation efficiency, and release profile.

**Results:**

The particle size was 147.2 nm, with 22.1 positive zeta potential and 96.8% encapsulation efficiency. The NPs released 45.6% of the encapsulated TQ within 3 h followed by characteristic sustained release over 7 days with a total of 69.7% cumulative release. TQ-PLGA NPs were taken up effectively by the cells in a time-dependent manner up to 24 h. Higher cell toxicity was determined within the first 24 h in melanoma cells due to the rapid release of TQ from the NPs and its low stability in the cell culture media.

**Conclusion:**

TQ-PLGA NPs is a potential anticancer agent taking advantage of the sustained release and tailored size that allows accumulation in the cancer tissue by the enhanced permeability and retention effect. However, stability problems of the active ingredient were address in this study and requires further investigation.

## Introduction

Malignant melanoma is the third most common malignant skin cancer and the most aggressive in terms of local invasiveness and mortality rate.^1^,^2^ A considerable attention is required in this malignancy not only due to the increase in its incidence rate,^1^,^3^ but also because this skin malignancy is characterized by its aggressive behavior that often cause treatment relapse with high mortality rate.^3^

*Nigella sativa* is well-known traditional medicine in the middle east that is used to treat chronic cardiovascular, hepatic, and renal diseases.^4^ Most of the pharmacological properties in this seed is attributes to its quinine derivative known as thymoquinone (TQ).^5^ TQ has been well known for its diverse therapeutic properties including antimicrobial, antioxidants, and anti-inflammatory effects. In cancer, it is deemed therapeutic through its interference with cell proliferation, apoptosis, cell invasion, and metastasis in cancer cells using different in vitro and in vivo models.^6^–^9^ Theses anticancer effects were reported in various types of cancers such as breast cancer cells,^10^ colon cancer,^11^ lung cancer,^12^ cervical squamous carcinoma,^13^ ovarian cancer,^14^ prostate cancer cell,^15^ and osteosarcoma.^16^ In addition, synergistic therapeutic effects were elicited when TQ was given in combination with other chemotherapeutic agents.^8^,^17^–^21^ The anticancer properties are mostly attributed to its antioxidant properties with pieces of evidence pointing towards its interference with specific cancer signaling pathways such as the MAPK, PI3-mTOR, JAK-STAT3 and NF-κB.^22^–^25^

Poly lactic-co-glycolic acid (PLGA) is a FDA approved polymer known for its biomedical applications in drug delivery due to its versatility, biodegradability, and biocompatibility.^26^ It is used extensively to prepare microparticles and nanoparticles to deliver a wide range of therapeutic agents including active pharmacological molecules,^27^ peptides,^28^ and nucleic acids.^29^

Therapeutic nanocarriers had shown promising results in the treatment of cancer partly due to the enhanced selective uptake in cancer tissue through the tumor capillaries a property known as the enhanced permeability and retention (EPR) effect.^30^,^31^ This effect is due to the disruption in the integrity of tumor capillaries and the inflamed endothelial cells potentiating the extravasation of nanocarriers through the leaky vasculature and enhancing the targeting strategy of treatment.^30^,^31^ The advantage of the EPR effect could be enhanced by nanocarriers based on specific properties such as the size and the surface charge of the particles.^30^ Particles of 100–200 nm size with hydrophilic surfaces tend to exhibit an improved EPR effect, which is attributed to the increased residence time of nano carriers in the blood circulation.^32^,^33^

Therefore, this study aimed to formulate TQ-loaded PLGA nanoparticles (TQ-PLGA NPs) with the evaluation of its properties and uptake in human melanoma cancer cells. The formulation was prepared to have a particle size between 100 and 200 nm, characterized and evaluated for cell uptake and cytotoxicity using the A375 melanoma cancer cell line.

## Materials and Methods

### Materials

Thymoquinone (Sigma Aldrich; WGK, Germany), medium molecular weight chitosan (190–310 kDa, 75–85% deacetylated) and polyvinyl alcohol (PVA) (Sigma Aldrich; Saint Lois, USA). Tween 80, Acetone and dichloromethane (Merck; Darmstadt, Germany). Poly lactic-co-glycolic acid 5004 (Purasorb; Corbion Purac, Holland). Coumarin-6 (Sigma-Aldrich; Milwaukee, Wisconsin, USA). High glucose of Dulbecco’s modified Eagle medium containing phenol red, L-glutamine and sodium pyruvate (Nacalai Tesque Inc; Kyoto, Japan), fetal bovine serum (Tico Europe; South America), HEPES buffer (Gibco, Life Technology Corporation; Auckland, New Zealand), the penicillin-streptomycin antibiotic (Gibco Invitrogen; Auckland, New Zealand), Tryple E (Gibco, Fischer Scientific; Massachusets, USA). Tissue culture wares (American Type Cell Culture (ATCC); Manassas, USA), all organic solvents used were of HPLC grade. MTT [3-(4,5-Dimethyl-thiazol-2-yl)-2, S-diphenyltetrazolium bromide] (Merck; Darmstadt, Germany), and dimethylsulfoxide (DMSO) (Sigma-Aldrich, Darmstadt, Germany).

### Nanoparticle Preparation

The fabrication process was adopted from Doolaanea A. M.^34^ Briefly, 6 mL aqueous phase (1% w/v chitosan, 0.2% PVA and 0.8% w/v Tween 80) was added into the oil phase (10 mg of TQ and 60 mg of PLGA dissolved in 2 mL mixture of 20:80 v:v ethyl acetate (EA) and dichloromethane (DCM)). The aqueous and oil phases were sonicated using a probe sonicator (Qsonica Q700, Newtown, USA) to form an emulsion that was then dropped slowly into a dispersion medium of 24 mL water and left on a magnetic stirring for 2 h for complete solvent evaporation. The nanoparticles were then washed to remove the excess stabilizers and were collected through centrifugation. Nanoparticles were then re-suspended in 2 mL distilled water and evaluated for nanoparticle size, zeta potential, and encapsulation efficiency. The nanoparticles were lyophilized to form a powdered dosage form using Christ Freeze Dryer (Martin Crist Alpha 1–2LD Plus, Pocklington, UK).

For quantitative analysis using a flow cytometer and qualitative analysis of the cell uptake using the fluorescence microscope, coumarin-6 a fluorescence dye was used. The dye was added together with other excipients that make up the oil phase (0.1% coumarin-6) together with the organic solvent and PLGA.

### Nanoparticle Characterization

#### Particle Size and Zeta Potential

A volume of 20 µL of nanoparticle suspension was diluted in 2 mL of distilled water, then the particle size and zeta potential were measured before lyophilization by dynamic light scattering (DLS) using Zeta sizer Nano-S and Nano-Z from Malvern Instruments Ltd (Malvern, Worcestershire, UK). The values were expressed as median diameter (D 50%) and millivolt (mV), for particle size and zeta potential, respectively. The polydispersity index (PDI) of the particle size was also reported.

#### Loading Efficiency

Encapsulation efficiency was measured indirectly by quantifying the amount of total un-encapsulated TQ using high-performance liquid chromatography (HPLC) with a validated analytical method as reported before.^35^ Briefly, after nanoparticle fabrication, 2 mL of the nanoparticle suspension was collected and the supernatant was obtained by centrifugation. The HPLC analysis was performed using Shimadzu LC-20AT equipment (Shimadzu, Japan). A mixture of acetonitrile and water in the ratio of 60:40 was used as a mobile phase at a flow rate of 1 mL/min using Inspire C18 (4.6 x 250 mm, 5 µm) analytical column. The detection was performed at a UV wavelength of 254 nm using a diode-array detector. The encapsulation efficiency was calculated based on the Equation below (Eq.1):

(1) $${\rm{Encapsulation\,Efficiency\,}}\left({\rm{\% }} \right){\rm{ = }}{{{{\left[{{\rm{TQ}}} \right]}_{\rm{i}}}{\rm{ - }}{{\left[{{\rm{TQ}}} \right]}_{\rm{f}}}} \over {{{\left[{{\rm{TQ}}} \right]}_{\rm{i}}}}}{\rm{ \times 100 }}$$

where ${\left[{TQ} \right]_i}$is the initial TQ concentration used in the oil phase and ${\left[{TQ} \right]_f}$is the final TQ concentration in the aqueous phase.

#### Scanning Electron Microscopy

The nanostructure of the particles was observed under a scanning electron microscope (SEM; Zeiss, Evo 50, Germany). The samples were sputter-coated with gold before observation under the SEM. The highest magnification images were obtained to observe the freeze-dried nanoparticle prepared from the optimized formulation.

#### Release Profile

Five milligrams of the optimized freeze-dried nanoparticles were suspended in 5 mL of phosphate buffer saline (PBS pH 7.4) and incubated at 37°C. At predetermined time points (0 h, 1 h, 3 h, 21 h. 42 h, and 1 week), 1 mL of the release medium was removed, and the NPs were separated by centrifugation. Fresh PBS was added to replace the taken amount. TQ in the release medium was evaluated using HPLC as mentioned before.

#### Differential Scanning Calorimetry (DSC)

DSC analysis of individual components of the nanoparticle formulation (PLGA polymer, TQ, Tween 80, PVA and chitosan) and the physical mixture of polymer + drug (1:1) was performed using PerkinElmer DSC-7^®^ (PerkinElmer, Inc., MA, USA). Equal weight of about 5 mg of each sample was loaded into 40 µL standard aluminum crucibles then heated under continuous nitrogen purging (20 mL/min) at a heating rate of 10°C/min to 350°C. An empty crucible served as a reference.

#### Fourier-Transform Infrared Spectroscopy (FTIR)

TQ, PLGA, PVA, TQ-PLGA NPs were examined for FTIR spectra in the range of 400 to 4000 cm^−1^ at 4 cm^−1^ resolution (Frontier Optica, Perkin Elmer, Pittsburgh, Pennsylvania, USA).

#### Stability of the Nanoparticles

Three types of stability assays were performed on TQ nanoparticle formulation and TQ solution. The first assay was based on the observation for changes in the particle size, PDI, and zeta potential using Malvern Zetasizer Nano-S and Nano-Z (Malvern Instruments Ltd, Malvern, Worcestershire, UK). The physical stability study was performed 1 month after preparation of the TQ-loaded PLGA nanoparticle suspensions at four different storage temperatures (−40°C, −20°C, 5°C and 25°C).

The second stability assay involved the assessment of nanoparticles in the cell culture medium. Here, the nanoparticle suspensions were added to complete cell culture medium (DMEM) at different concentrations (0.1 mg/mL to 10 mg/mL). The nanoparticle suspensions were incubated at 37ºC and were tested after 24 h and 48 h, respectively. The parameters used included changes in the particle size, PDI, and zeta potential.

The third stability assay involved the assessment of chemical degradation of TQ in complete cell culture medium (DMEM) using the HPLC.

#### Cell Culture

A375 human melanoma cells were obtained from American Type Cell Culture (ATCC), Manassas, USA. The cells were cultured in T75 and T25 flasks (Eppendorf, San Diego California, USA) at 37°C and 5% CO_2_ using High glucose Dulbecco’s modified Eagle medium (DMEM) Eagle’s Minimum Essential Medium (EMEM). Fetal bovine serum, penicillin-streptomycin, and HEPES buffer were added to a final concentration of 10%, 2%, and 1%, respectively, to complete the growth medium.

#### Cell Uptake of Nanoparticles

A375 cells were seeded in 96-well flat plate at a concentration of 1 x 10^4^ cells/100 µL/well. For concentration-dependent studies of the cell uptake, after the cells adhered to plate within 24 h then treatment started with 0.1, 1.0, 2.5, 5, and 10 mg/mL concentrations of coumarin-TQ-loaded PLGA NPs for 24 h. Meanwhile, for time-dependent cell uptake, the cells were assessed after 2 h, 6 h, and 24 h of treatment, respectively, using a fixed concentration of the NPs. On the time of assay, the cells were washed gently with ice-cold PBS three times before being detached by triple E in the incubator for 2 min.^36^ The cells were centrifuged at 800 g for 4 min and the supernatant was discarded. The cells were then re-suspended in 4% of formaldehyde for cell fixation and incubated in ice for 10 min. The cells were centrifuged and re-suspended in PBS buffer saline before being evaluated with the flow cytometer. The forward light scatters (FSC) were used to count the total number of cells after the deduction of the background caused by some free NPs in the medium. On the other hand, the green fluorescence channel was used to count the positive cells (successful cell uptake if fluorescent NPs).

The efficiency of cell uptake was also qualitatively assessed using a fluorescent microscope (Cytell cell imaging system, GE Healthcare life science, Buckinghamshire, UK). In this assay, the A375 cells were seeded on round-coated coverslip mounted in 24-well flat plate at a concentration of 1 x 10^4^ cells/well. Cells were allowed to attach for 24 h and then the cells were incubated with a suspension of 1.0 mg/mL coumarin-loaded nanoparticles in growth medium for 2 h. The coverslips were then transferred into wells containing ice-cold 4% paraformaldehyde for 10 min fixation. Then the coverslip was lifted and placed on top of the glass slide with cells facing upwards. A 50 µL of mounting medium containing fluorescent dyes of DAPI and phalloidin with the ratio 1:1 was dropped on the cells before being covered by square coverslip cleaned with 70% ethanol. The glass coverslips were sealed to retain the position on the glass slide before being examined under the Cytell fluorescence microscope.

### In vitro Cancer Cytotoxicity Assay

The cytotoxic effect of TQ solution and NPs form was evaluated using the MTT assay (3-(4,5-dimethylthiazol-2-yl)-2,5-diphenyltetrazolium bromide assay) in the A375 cells. The cells were seeded in 96-well flat plate at a concentration of 1 x 10^4^ cells/well and allowed to attach for 24 h. The cells were then treated with TQ solution, TQ-PLGA NPs and blank NPs using concentration gradients of (1–100 µg/mL) and (0.1–10 mg/mL) respectively for 24 h and 48 h. After the treatment, the medium was aspirated, and the cells were washed with PBS and treated with MTT reagent (5 mg/mL in PBS). The formazan crystals produced by mitochondrial reductase enzyme of the living A375 cells were dissolved using dimethyl sulfoxide (DMSO) with continuous agitation. The cells were incubated at room temperature for 30 min before measuring the light absorbance at 570 nm wavelength using a microplate reader (Infinite M200 Nanoquant, Tecan, Austria). The percentage of cell viability were calculated, by deducting the absorbance of the cell-free wells containing MTT only from the absorbance values of treatment wells and then dividing the corrected absorbance value on by the absorbance of healthy-untreated cells.

### Statistical Analysis

Comparisons amongst the three formulations were carried out using analysis of variance (ANOVA) with Tukey’s post hoc test while *t*-test was employed for comparisons within each formulation. P-value < 0.05 was considered statistically significant. Minitab software version 16 (Minitab, State College, PA) was used to perform the statistical analysis.

## Results and Discussion

### Characterization of Nanoparticles

**Figure 1:**
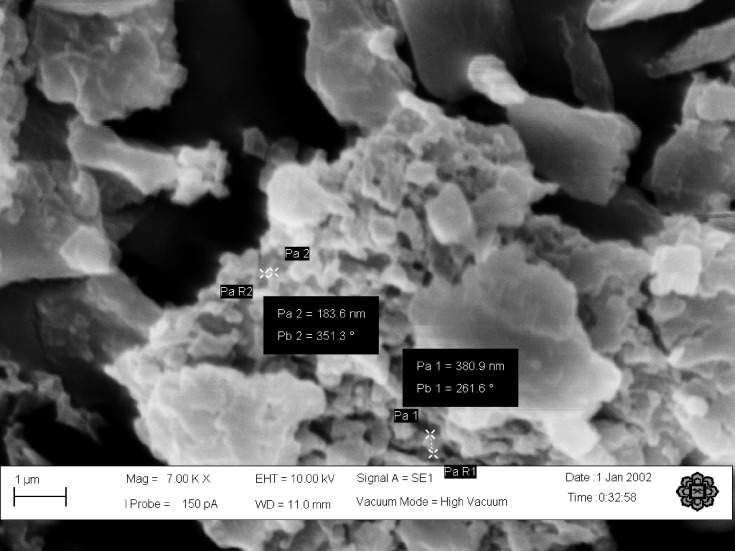
SEM image of the freeze-dried TQ-PLGA NPs.

TQ-PLGA NPs exhibited monomodal particle size distribution (Figure 1) with an average size of Di50 = 147.2 ± 0.4 nm with PDI of 0.142 ± 0.017. The NPs showed a positive zeta potential of 22.1 ± 1.1 attributed to the adsorption of chitosan on the surface. TQ encapsulation efficiency was high (96.81 ± 0.05%) thanks to its low water solubility. The solubility of TQ was reported as 549–669 μg/mL in the aqueous solutions.^37^ High encapsulation efficiency is usually obtained with hydrophobic drugs when using the solvent evaporation method for nanoparticle preparation. The low aqueous solubility prohibits the material from escaping to the external phase. This is applicable to TQ which is a hydrophobic material with low aqueous solubility.

**Figure 2:**
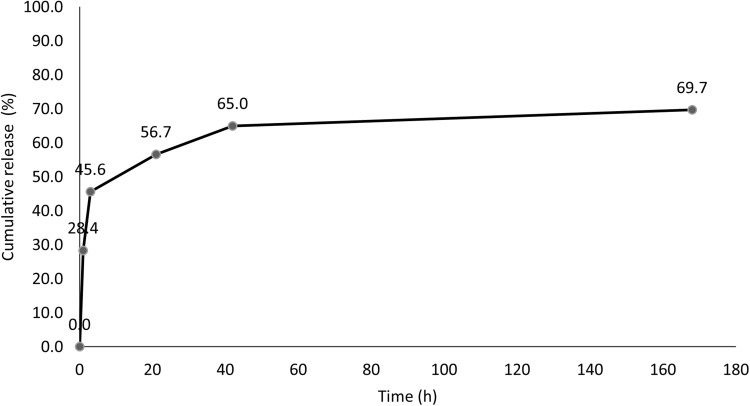
Release profile of the optimized TQ-PLGA NP formulation (Mean ± SE, n = 3). The release profile has two phases; burst release within 24 h followed by sustained release up to one week.

The *in vitro* release of TQ from PLGA NPs showed a typical biphasic release pattern (Figure 2). The NPs released 45.6% of the encapsulated TQ within the first 3 h followed by characteristic sustained release. The cumulative percentage release of TQ was 65.0% within 48 h followed by slower release pattern to reach only 69.7% after 1 week. The initial burst of TQ release was likely due to the release of TQ loosely attached to the surface of the nanoparticles, whereas the later slow release may indicate the release from the of PLGA NPs matrix.

**Figure 3:**
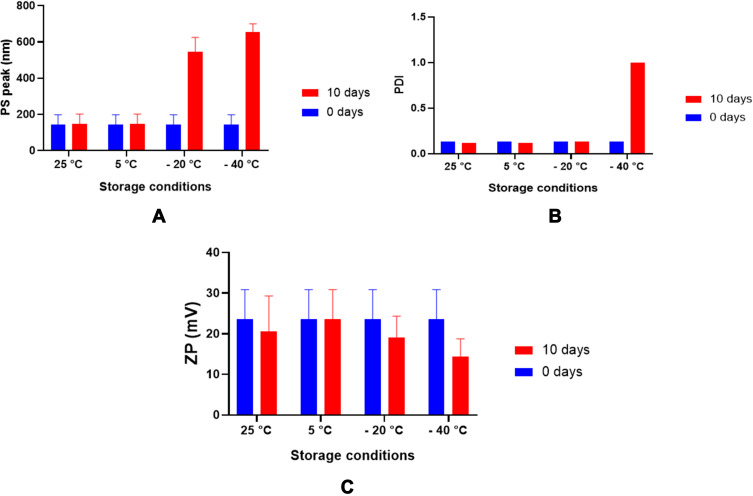
Stability of TQ-PLGA NP suspension at four different storage conditions. The stability indicators included (**A**) PS (particle size), (**B**) PDI (polydispersity index) and (**C**) ZP (zeta potential).

Optimum storage conditions for TQ-PLGA NPs were evaluated in this study in which the particles were stored in different storage conditions for 10 days. At freezing temperatures of −20°C and −40°C, the nanoparticles demonstrated a propensity to aggregate after defrosting the nanoparticle suspension that was indicated by the increase in particle size (Figure 3). This may be attributed to the detachment of chitosan molecules from the surface of the nanoparticles as indicated by the decrease of zeta potential values. These loosely attached or detached chitosan molecules may contribute in the adherence between adjacent nanoparticles causing aggregation. When TQ nanoparticles were prepared without using chitosan, the suspension displayed significantly better solubility and less aggregation which further support the claimed observation in this study.^36^ Therefore, uncoated PLGA NPs exhibited better suspension stability than chitosan-coated PLGA NPs at freezing temperatures.

The nanoparticles structure plays an important role in determining their interaction and adhesion with body cells. To determine the morphology of the nanoparticles, SEM was carried out. The nanoparticles appeared as clumps of grainy like structure that is mostly attributed to the effect of freezing on the nanoparticle suspension as shown (Figure 3). This finding was supported in other studies, where larger particles are produced when using rapid freezing rate.^38^ The tendency of nanoparticles for aggregations are more likely to happen when the van der Waals attractive forces between nanoparticles are larger than the electrostatic repulsive forces during the freezing process.^39^ Chitosan and PVA provide steric stabilization through the binding on the surface of the nanoparticles while Tween 80 provides electrosteric stabilization to the nanoparticle suspension.^40^ Coating TQ-PLGA NPs with these three materials may have contributed in producing the weak steric or electrostatic stabilization of the nanoparticle rendering most of the particles in a fragile state that could collapsed easily upon freezing and thawing.

The nanoparticles formulations in powdered form are usually more stable than being in the suspension form. Thus the powdered form may prevent the premature release of the encapsulated drug in the dispersed phase; it therefore allows longer terms of storage and ease of transport.^41^

The aggregation problem observed in the study could be overcome by the usage of cryoprotectants as they shield the nanoparticles from one another through particle isolation hypothesis and therewith hinder the aggregation.^42^ Based on the findings in the study it is recommended to store TQ-PLGA NPs in the fridge (5 °C) and use the particles suspension within 10 days.

**Figure 4:**
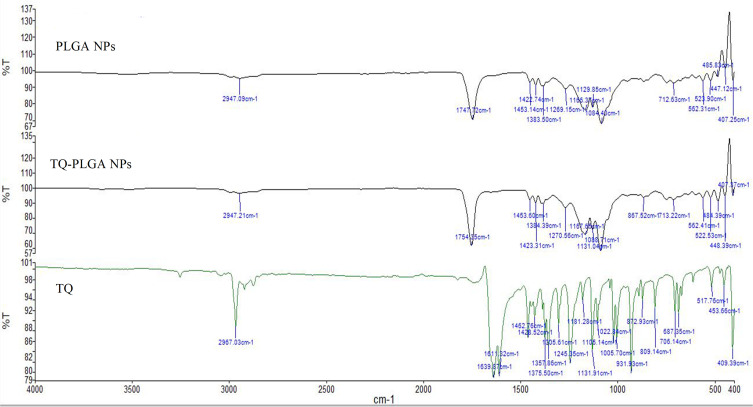
ATR-FTIR spectra of PLGA NPs, TQ-PLGA NPs, and TQ.

FTIR spectra helps to study interactions between different components in the formulation and such interactions are indicated by appearance or disappearance of peaks or peaks shift.^43^ The infrared spectra of TQ, blank nanoparticles (PLGA NPs), and TQ-PLGA NPs are shown in Figure 4. The characteristic spectra of all of the samples showed the alkane groups (–CH, –CH2, –CH3) stretching at 2800–3000 cm^−1^, and ethers group (C–O) stretching at 1050–1250 cm^−1^. For both blank and loaded nanoparticles, the carboxyl group (–C=O) of PLGA exhibited stretching at 1750–1625 cm^−1^. Peaks in range of 1600–1580 cm^−1^ are attributed to the benzene ring of TQ. The characteristic peaks in TQ-PLGA NPs were identical to those of blank PLGA NPs and were therefore attributed to the peaks of PLGA polymer. On the other hand, TQ peaks were not detected in the loaded TQ-PLGA NPs probably due to the small percentage of TQ compared to PLGA and the weak absorption bands of TQ compared to PLGA.

**Figure 5:**
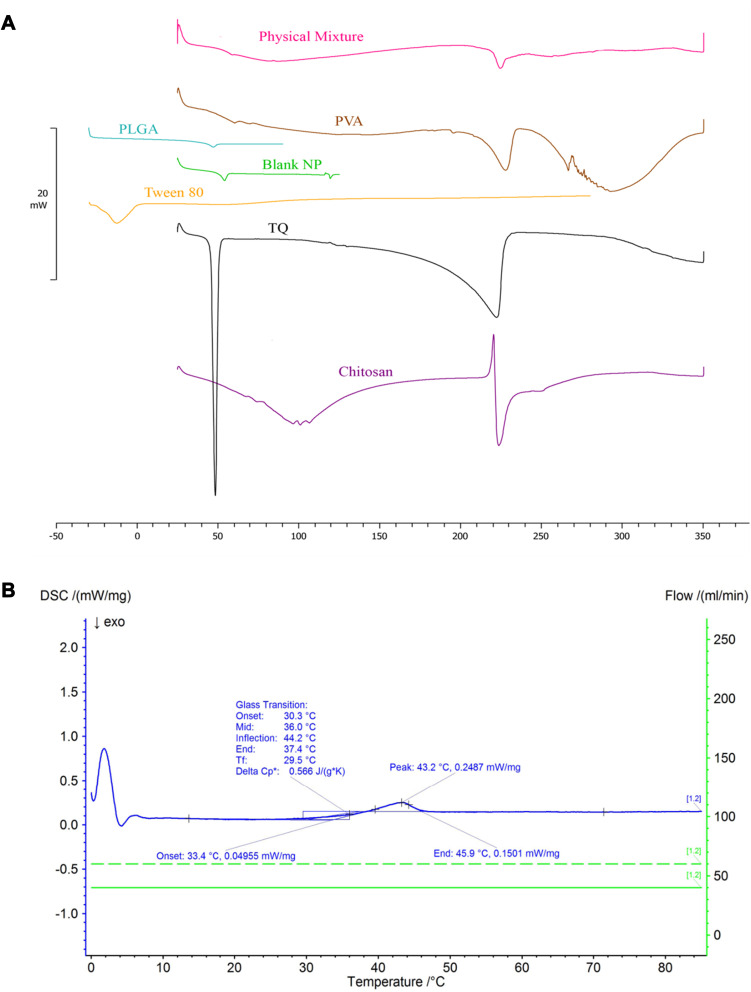
DSC thermograms of (**A**) TQ and the components in the NP formulation, physical mixture, and blank NPs and (**B**) TQ-PLGA NPs.

Differential scanning calorimetry (DSC) thermograms for the NP components, physical mixture, blank NPs, TQ, and TQ-PLGA NPs are shown in Figure 5. Melting temperature T_m_ of TQ was found at 48.33°C. PLGA polymer showed a glass transition T_g_ onset at 42.45°C with clear enthalpy relaxation. There was no shifting in T_g_ of the blank nanoparticle curve. TQ-PLGA NP T_g_ onset was at 30.3°C suggesting a remarkable plasticizing effect of TQ on PLGA. Lowering the glass transition below body temperature may contribute to the rapid release of TQ from the nanoparticles. This is due to the increase in polymer molecular mobility above the glass transition temperature. Consistent observations were reported in TQ, PLGA, and physical mixture thermograms of other studies.^44^

TQ melting peaks disappeared in the thermogram of TQ-PLGA NP denoting the amorphous state of TQ in the NP formulation. This may contribute to the fast release from the nanoparticles as amorphous forms usually have better solubility than their crystalline counterparts.

### Stability of Nanoparticles in Cell Culture Media

**Figure 6:**
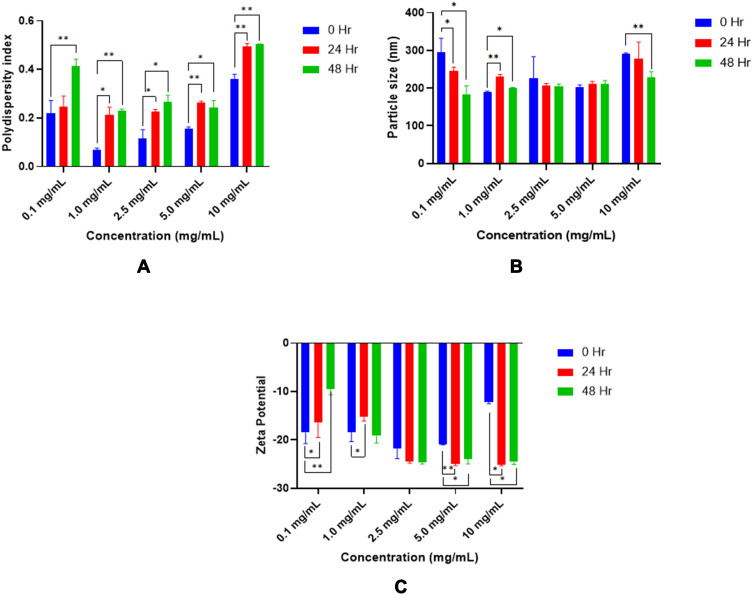
TQ-PLGA NP stability in DMEM media in term of polydispersity index (**A**), particle size (**B**), and zeta potential (**C**) incubated at 37ºC. The values are mean ± standard deviation, n=3, *t*-test of variance, *p-value ≤0.05, **p-value ≤0.001.

The physical stability of TQ-PLGA NPs was evaluated by measuring particle size, PDI, and zeta potential of various nanoparticle concentrations in the media used for the cell viability, cell cytotoxicity, and cell uptake studies at 37ºC as summarized in Figure 6.

As shown, there was no significant change in the particle size within 24 h (p > 0.05); however, after 48 h significant changes were elicited as indicated in the figure (p < 0.05).

The poly disparity index (PDI) of the of TQ-PLGA NPs demonstrated significant changes in culture media after 24 h and 48 h storage at different NP concentrations. While the zeta potential values remained considerably stable with lower NP concentrations, unlike the changes observed at higher concentrations. These changes are probably due to the adsorption of protein components within the media with the NPs. The colloidal stability mainly depends on the electrical double layer and the steric repulsion of the particles. The components in the cell culture media like serum albumin/globulins, amino acids, and ionic salts influence the hydrodynamic size and the charge of nanoparticles and these constituents may destabilize the nanoparticle suspension by adsorption leading to loss of surface integrity and function leading to profound aggregation by the electrostatic interactions.^40^

**Figure 7:**
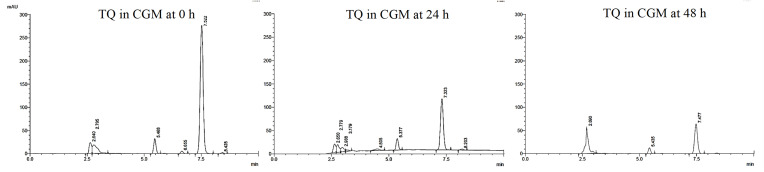
HPLC chromatograms demonstrating the stability of TQ in complete cell culture media at 0, 24 and 48 h incubation (37 °C).

In addition, TQ degradation in the complete growth media was evaluated at the optimum culture conditions at 37ºC using HPLC analysis as represented in Figure 7. As shown, approximately 18.0% and 77.0% of degradation occurred within 24 h and 48 h, respectively. Even though TQ exerts a considerable rate of degradation within 48 h in CGM at 37ºC, the use of PLGA as carrier played an important role in increasing the stability of TQ and enhance its bioavailability for safe delivery to the targeted cells.^45^

### Cellular Uptake of the Nanoparticles

**Figure 8:**
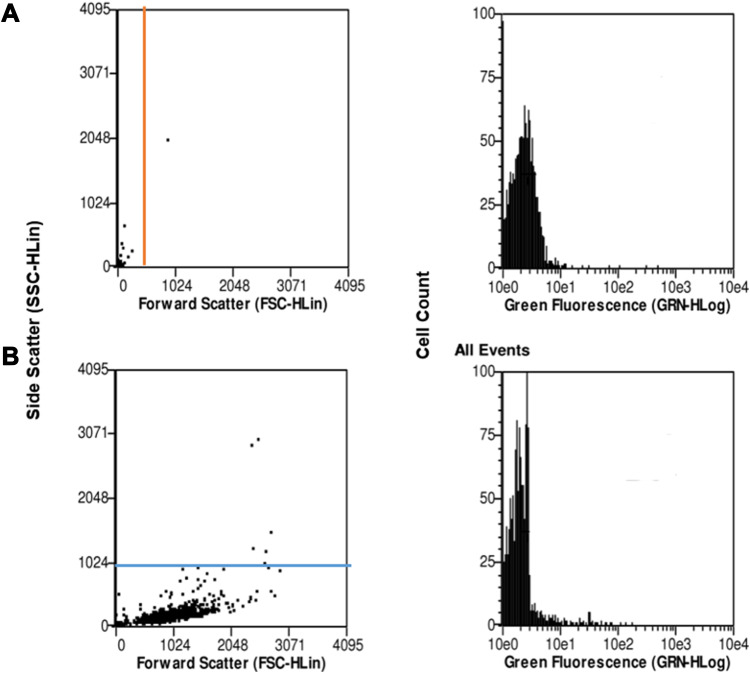
Flow cytometric analysis of the controls (**A**) free nanoparticles spiked in a media (positive control) and (**B**) untreated cells (negative control).

**Figure 9:**
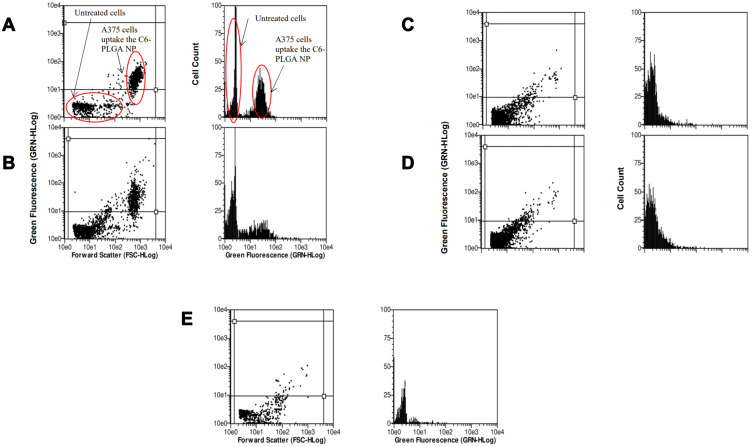
Flow cytometric analysis of A375 melanoma cells when incubated with coumarin-loaded nanoparticles in different concentration of nanoparticles suspension; (**A**) 0.1 mg/mL, (**B**) 1.0 mg/mL, (**C**) 2.5 mg/mL, (**D**) 5.0 mg/mL and (**E**) 10.0 mg/mL incubated for 24 h.

**Figure 10:**
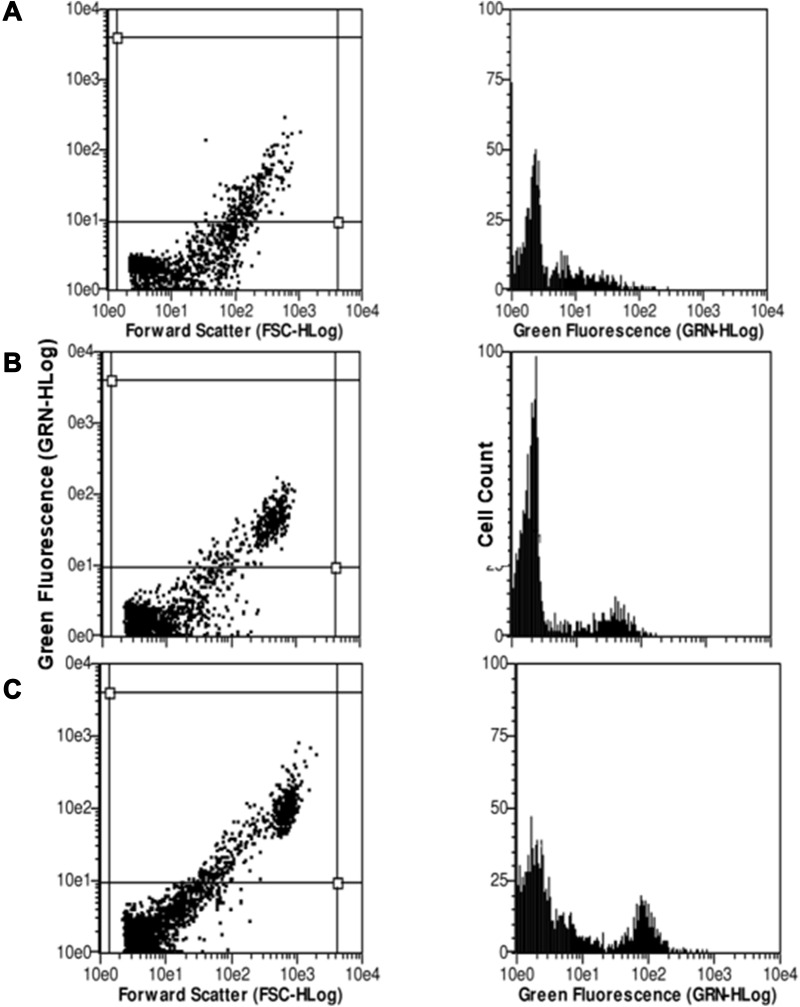
Flow cytometric analysis of A375 melanoma cells when incubated with coumarin-loaded nanoparticles treated with 1.0 mg/mL concentration of nanoparticles suspension at 3 different time points; (**A**) 2 h (**B**) 6 h, and (**C**) 24 h.

**Figure 11:**
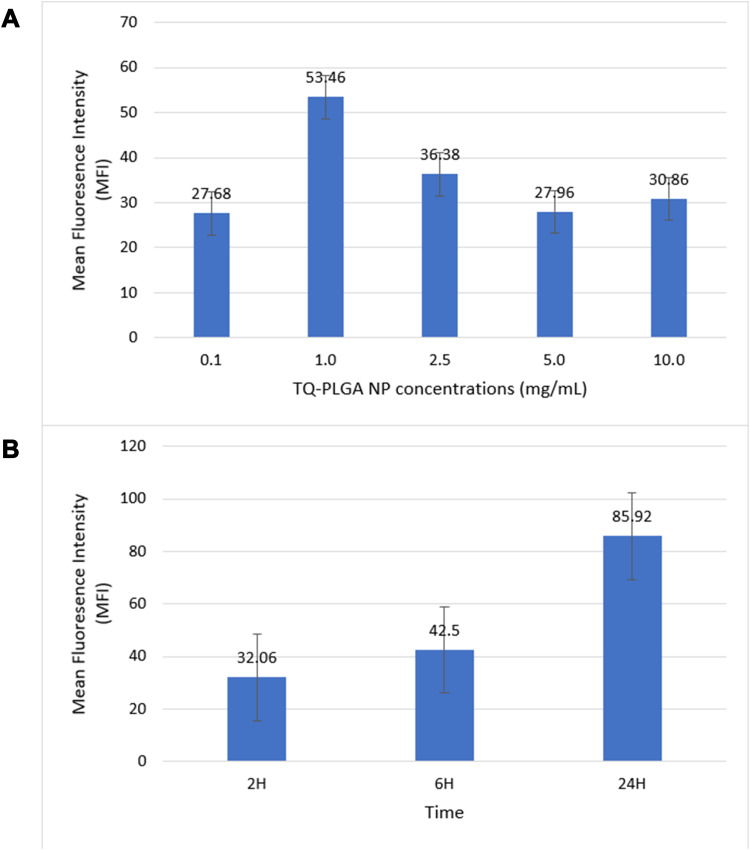
Cellular uptake mean fluorescence intensity (MFI) of coumarin-6 nanoparticles (C6-PLGA NPs) by A375 cell line at (**A**) different concentrations of nanoparticles and (**B**) three different times of incubation.

The cellular uptake of TQ nanoparticles was evaluated based on the principles of flow cytometry in which the forward-scattered light (FSC) is proportional to the cell size whereas the side-scattered light (SSC) is related to cell internal complexity.^46^ The color detection throughout this study was the green color emitted by coumarin-6 fluorescent dye. In the beginning, parameters were set to exclude the free NPs (not being taken up) and the background fluorescence of the untreated A375 cells as shown in Figure 8. Despite washing with ice-cold PBS to remove the non-internalized NPs, some nanoparticles were still present as shown in the Figure. These free nanoparticles exhibited high fluorescence with low forward scattering due to their small size compared to the cell size. Meanwhile, the cell that did not take up nanoparticles had lower fluorescence emissions. Therefore, the vertical line at value 12 on the forward scattering x-axis was set to exclude the background fluorescence of free nanoparticles while the horizontal line at value 100 on the fluorescence y-axis was set to exclude the background fluorescence from the untreated A375 cells. This comparable exclusion criterion was also used in other reports.^47^ The evaluation of cell uptake using the flow cytometry are demonstrated as histogram plots in Figure 9 for concentration-dependent assessment and Figure 10 for time-dependent assessment. Figure 11 shows the quantification data of coumarin-6 (C6)-PLGA NP uptake by the A375 cells based on the mean fluorescence intensity. The highest intracellular uptake of C6-PLGA NP was at 1.0 mg/mL with time-dependent increase in cell uptake up to 24 h.

The study confirmed that the size and surface charge of the nanoparticles had contributed significantly to cellular uptake. The positive surface charge of the NPs interacted with the negatively charged cell membrane of the A375 cells facilitating the internalization of the C6-PLGA NPs. This finding is supported by the reported effect of PLGA-nanoparticles that quickly escapes the endo-lysosomes and were efficiently internalized within the cell cytoplasm after 10 min incubation time.^48^ This was attributed to the interaction between the nanoparticles and the vesicular membranes leading to transiently localized destabilization of the cell membrane resulting in the escape of nanoparticles into the cytosol.

The highest intracellular uptake was at 1.0 mg/mL concentration. Higher concentrations had considerably decreased the cellular uptake probably because of NPs aggregation in the complete growth medium as confirmed by the physical stability assay shown in Figure 6. In addition, the changes in the PDI of the nanoparticles were more obvious at the higher NP concentrations. The demonstrated NPs aggregation was more evident at high concentration (10 mg/mL); whereby the measured particle size in the complete growth medium was 291 nm compared to 189 nm at 1 mg/mL concentration. This explains how smaller particles are taken up at higher extent in biological cells compared to larger particles as evidenced by reports.^49^

At 1 mg/mL concentration, the time-dependent cell uptake demonstrated a proportional increase with time up to 24 h. This is consistent with other studies that reported the positive correlation between cell uptake and time until it reached a plateau or saturation.^50^,^51^ This may highlight the importance of the extending the nanoparticles availability in the blood circulation or in the cancer tissue to enhance the internalization within cancer cells.

**Figure 12:**
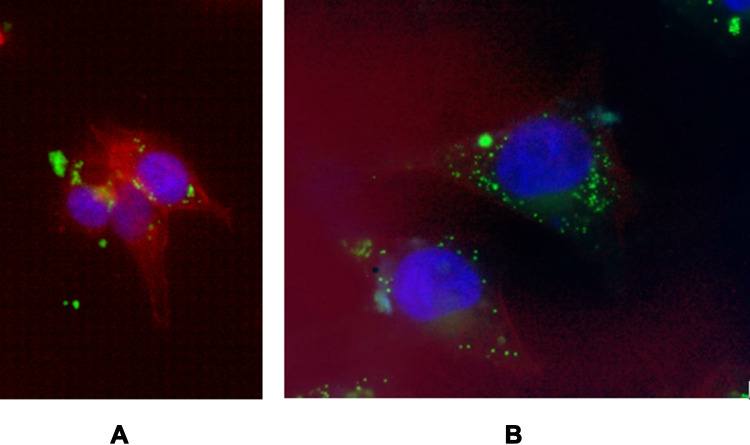
Cellular uptake of coumarin-6 nanoparticles (C6-PLGA NPs) by A375 cell line as seen under fluorescence microscope after immunofluorescent staining of the actin cytoskeleton and the nucleus of the cells using TRITC-conjugated phalloidin (red) and DAPI (blue) respectively; (**A**) 40X, (**B**) 100X magnifications.

The internalization of C6-PLGA nanoparticles into A375 cells was also visualized using fluorescence microscopy using 1.0 mg/mL concentration of C6-PLGA NPs for 2 h (Figure 12). Coumarin-6 is considered suitable for cell uptake studies since it does not cause acute toxicity and is found stable post encapsulation with no instant release into the cellular media once internalized.^52^ As shown, the C6-PLGA NPs were seen inside the A375 cells and well distributed into the cytosol while some were allocated around the nucleus. The images also confirmed the average size of the NPs though there are some aggregated NPs that could be seen.

### In vitro Anticancer Activity of TQ and TQ-PLGA NPs

One of the properties of cancer tissues is that it tends to have a highly permeable vasculature allowing nanoparticles with a size of about 100–200 nm to pass easily and accumulate in the cancer tissue as described earlier. This allows the nanoparticle administered through the intravenous route to be passively delivered to the targeted cells.^30^,^31^ TQ-PLGA NP formulation had a size of 147.2 nm, that was evaluated for its cytotoxic properties in A375 melanoma cancer cells in comparison with the TQ solution.

**Figure 13:**
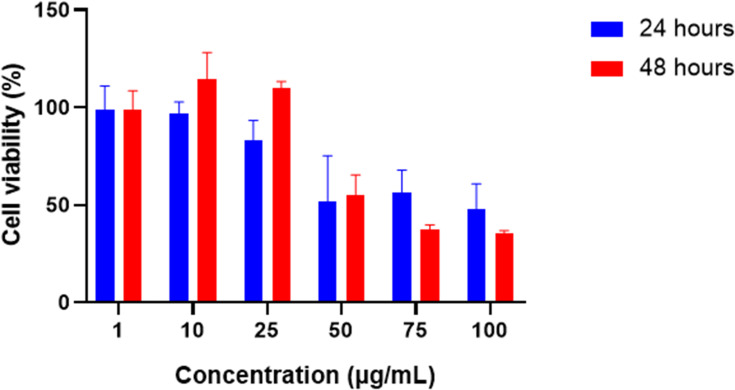
Cell viability of A375 cells treated with different concentrations of TQ solution for 24 h and 48 h.

The cytotoxicity assessment of TQ solution in A375 cancer cells was determined at two-time intervals (24 h and 48 h) using different concentrations as shown in Figure 13. The figure demonstrates a dose-dependent cytotoxic property of TQ solution that reached a significant level at concentration of 50µg/mL, where the proliferation of melanoma cancer cells was inhibited by approximately 50%. As shown, the cytotoxic effects were significantly lower after 48 h treatment probably due to the low stability and degradation of TQ in the culture media. As such, a higher trend of TQ cytotoxicity was elicited among the cancer cells within the first 24 h of incubation. Low concentrations of TQ had not shown any inhibitory effect where the cells displayed nearly a 100% viability after 24 h treatment. This may be attributed to rapid proliferation of A375 cells with reported doubling time of 6–12 h in the first 24 h that is consistent with other reports.^53^

As demonstrated after 50µg/mL concentration of TQ, the dose-dependent response reached a plateau at approximately 50% cell viability. The IC_50_ concentration of TQ-solution in A375 cells was between 50 µg/mL and 100 µg/mL with 48.0% and 51.1% at 24 h, and 35.6% and 55.1% at 48 h, respectively. The reported IC_50_ concentration of TQ was significantly higher than other studies conducted on melanoma cells lines and this difference may be attributed to difference of cell line virulence and proliferation.^54^

**Figure 14:**
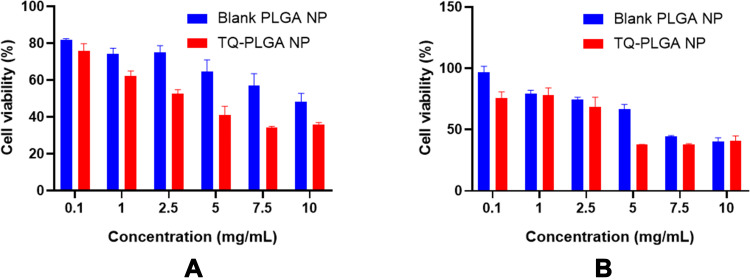
Cytotoxicity study (MTT assay) of blank nanoparticles and TQ-PLGA NPs in A375 human melanoma cancer cells. (**A**) 24 h and (**B**) 48 h.

In the developed formulation of TQ (TQ-PLGA NP), the A375 cells showed a concentration-dependent cytotoxic effect as shown in Figure 14. As shown, the cytotoxic effects of TQ-PLGA NPs reached its maximum rate at the concentration of 7.5 mg/mL with 34.2% cell viability within 24 h of treatment.

The blank nanoparticle suspension had also demonstrated cytotoxic effects in cancer cells as the viability decrease to 40.3% at the highest concentration of the nanoparticles after 48 h of incubation. Based on other observations, PLGA nanoparticles of 200 nm size are unlikely to exhibit cytotoxic effects at concentration range of 10 µg/mL to 300 µg/mL.^55^,^56^

The observed effect of blank nanoparticles may be attributed to particles aggregation at higher concentrations imposing more toxicity among the cells.^57^ The positively charged NPs tend to aggregate more due to the bridging flocculation between negatively charged proteins and positively charged NPs.^40^

Based on the release profile of the optimized nanoparticle formulation, 56.7% of the encapsulated TQ was released from the nanoparticles within the first 24 h. therefore less cytotoxic effects were observed after 24 h due to the slower release of TQ from the drug carrier. TQ-PLGA NPs had considerably higher cytotoxicity than blank nanoparticles in the first 24 h incubation. However, the difference was not significant after 48 h which can be attributed to the rapid release (~56.7%) of TQ in the first 24 h and the degradation of TQ in the culture media (77.0% after 48 h).

The IC_50_ concentration of TQ-PLGA NPs in A375 cells was between 2.5 mg/mL and 5 mg/mL with 41.0% and 52.6% cell viability at 24 h and 37.5–68.3% cell viability at 48 h, respectively. The highest cytotoxic effects of the developed NPs in the A375 cells were achieved at 7.5 mg/mL concentration at 48 h.

As shown in the figure, higher concentrations of TQ were required to produce the same cytotoxic effects when encapsulated in PLGA NPs. The privileges of using this formulation despite the higher concentration include the added advantages of nanocarriers in vivo settings when compared with free TQ solution. The nanocarriers will contribute in increasing the bioavailability of TQ by enhancing aqueous solubility, increasing the half-life in the circulation, and enhance the targeted accumulation of nanoparticles within cancer tissue.^30^,^58^

## Conclusion

In this study the TQ-loaded PLGA nanoparticles (TQ-PLGA NPs) were successfully prepared with a particle size of 147.2 nm with a positive zeta potential and high encapsulation efficiency. The TQ-PLGA NPs were taken up effectively by the cancer cells in a time-dependent manner up to 24 h. The optimum cell uptake and cytotoxicity of the TQ-PLGA NPs in cancer cells were challenged by the stability of TQ, aggregation and the rate of release. TQ stability in the cell culture media was rarely considered in previous studies and our study highlights the significance of TQ stability in the treatment medium. The challenges of TQ stability in aqueous solutions and the demonstrated cytotoxic effects in cancer cells demand further investigation with extrapolation to more in vitro and in vivo experiments considering its application as a cancer chemotherapeutic agent.
